# Maternal Overnutrition and Fetal Programming: Long-Term Metabolic, Cognitive, and Epigenetic Consequences

**DOI:** 10.3390/cells15040366

**Published:** 2026-02-18

**Authors:** Gabriella Schiera, Giulia Macajone, Sara Volpes, Laura Greco, Carlo Maria Di Liegro, Graziella Serio, Fabio Caradonna, Flores Naselli

**Affiliations:** 1 Department of Biological, Chemical and Pharmaceutical Sciences and Technologies (STEBICEF), University of Palermo, Viale Delle Scienze Building 16, 90128 Palermo, Italy; gabriella.schiera@unipa.it (G.S.); giulia.macajone@community.unipa.it (G.M.); sara.volpes@unipa.it (S.V.); laura.greco08@unipa.it (L.G.); carlomaria.diliegro@unipa.it (C.M.D.L.); graziella.serio01@unipa.it (G.S.); fabio.caradonna@unipa.it (F.C.); 2 NBFC, National Biodiversity Future Center, 90133 Palermo, Italy

**Keywords:** maternal obesity, epigenetic inheritance, fetal programming, transgenerational epigenetic effects, gestational diabetes mellitus

## Abstract

Maternal nutrition during pregnancy critically influences fetal programming, shaping the offspring’s lifelong health and disease susceptibility. Both undernutrition and overnutrition affect fetal metabolism, predisposing offspring to obesity and cardiometabolic disorders in adulthood. This review examines current evidence on how maternal nutrition, particularly overnutrition and its complications, such as gestational diabetes mellitus (GDM) and obesity, affects offspring health. It also explores the biochemical and epigenetic mechanisms underlying aberrant fetal programming induced by an unfavorable intrauterine environment. Excess nutrient exposure in utero alters fetal metabolic pathways by modifying the expression of key metabolic genes and nutrient sensors, increasing susceptibility to metabolic syndrome later in life. Maternal obesity has additionally been linked to cognitive dysfunction, immune alterations, and elevated cancer-related mortality in the offspring. GDM exposure disrupts fetal hypothalamic development, impairing appetite regulation. Emerging evidence suggests that epigenetic changes induced by maternal overnutrition may be transmitted across generations and that paternal obesity may also contribute to fetal metabolic programming. Although lifestyle interventions during pregnancy have been tested, they show limited long-term benefits, whereas pre-pregnancy BMI remains the strongest predictor of offspring obesity, emphasizing the critical role of preconception care and the prevention of overweight in women of reproductive age.

## 1. Introduction

The Developmental Origins of Health and Disease (DOHaD) hypothesis, first articulated by Barker and colleagues in the late 20th century, provides a conceptual framework for understanding how environmental exposures during critical periods of development shape long-term health trajectories [1,2]. According to this paradigm, the intrauterine environment acts as a crucial interface between maternal physiology and fetal development, influencing cellular differentiation, organogenesis, and metabolic programming. The fetus exhibits a remarkable degree of plasticity, enabling physiological adaptations to adverse intrauterine conditions, such as nutrient deprivation or hormonal imbalances. However, such adaptations—while advantageous for short-term survival—may predispose the individual to metabolic, cardiovascular, and neurodevelopmental disorders later in life, a phenomenon often referred to as “fetal programming” [3,4].

While nutrition represents a primary determinant of developmental programming, it operates within a complex matrix of environmental exposures. Maternal psychosocial stress, environmental pollutants, and endocrine-disrupting chemicals can interact synergistically with nutritional factors to modulate epigenetic programming and metabolic outcomes. For instance, prenatal stress may alter maternal eating behaviors and nutrient partitioning, while exposure to heavy metals and persistent organic pollutants can interfere with micronutrient metabolism, potentially amplifying the adverse effects of nutritional imbalances on fetal development [5].

A growing body of evidence suggests that these developmental effects are mediated by epigenetic mechanisms, which collectively influence gene expression patterns without altering the DNA sequence [6,7]. These heritable molecular signatures provide a mechanistic basis for the transgenerational transmission of disease risk associated with suboptimal intrauterine environments. Inadequate maternal intake of essential micronutrients—including folate, iron, iodine, zinc, and vitamin D—has been associated with increased risks of intrauterine growth restriction (IUGR), neurodevelopmental impairment, and long-term metabolic dysfunction in the offspring [8,9]. A comprehensive understanding of the significance of maternal nutrition was first highlighted by observations following the Dutch Famine or Hunger Winter (1944–1945), a dramatic historical event. Individuals whose gestation coincided with the famine exhibited increased susceptibility to obesity, insulin resistance, and cardiovascular disease in adulthood, strongly implicating fetal adaptive responses to nutrient scarcity in shaping lifelong disease risk [10,11].

While undernutrition remains a pressing issue in several developing regions, maternal overnutrition and obesity have emerged as major public health challenges in industrialized societies. These conditions, often accompanied by relative micronutrient imbalances, expose the fetus to an environment of nutrient excess and hyperinsulinemia, promoting excessive fetal growth, altered adipogenesis, and increased risk of metabolic disorders later in life [12,13]. Thus, the maternal malnutrition spectrum represents a critical determinant of developmental and metabolic programming.

**Figure 1:**
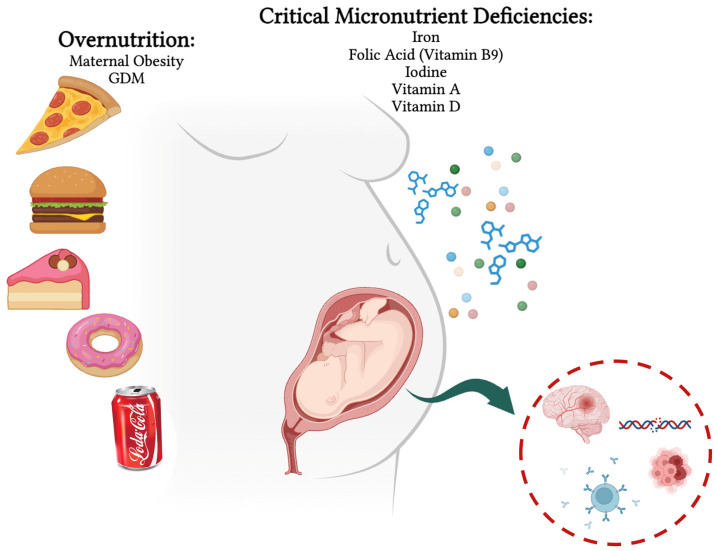
Schematic representation of the effects of maternal overnutrition and micronutrient imbalance on offspring health outcomes. The food items shown are intended to represent an ‘unhealthy diet’ during gestation. Specifically, we have included items rich in saturated fats and sources of high sugar intake, which commonly contribute to maternal hyperglycemia. Original figure created by the authors using Microsoft PowerPoint Office 365.

The objective of this review is to provide an integrated synthesis of the long-term metabolic, cognitive, and epigenetic outcomes arising from maternal overnutrition, GDM, and micronutrient status during pregnancy. Beyond summarizing epidemiological and physiological evidence, this review elucidates the cellular and molecular mechanisms underlying these phenomena, offering insights into how intrauterine nutritional exposures are transduced into persistent phenotypic changes in the offspring. Emphasis will be placed on epigenetic modifications as mechanistic mediators linking maternal diet and metabolism to altered fetal gene expression [14,15,16]. These epigenetic marks may interact with biochemical and signaling pathways, including mTOR, AMPK, PPARγ, and insulin/IGF signaling, leading to persistent changes in energy homeostasis, mitochondrial function, oxidative stress responses, and neurodevelopmental plasticity [17,18]. Therefore, this review aims to provide an integrated mechanistic synthesis of the long-term metabolic, cognitive, and epigenetic consequences of maternal overnutrition, gestational diabetes mellitus, and micronutrient imbalance, uniquely linking intrauterine nutritional exposures to persistent offspring phenotypes through epigenetic regulation and key metabolic signaling pathways (Figure 1).

## 2. Maternal Metabolic Adaptations and Nutritional Status

Pregnancy is a physiological process bringing about significant changes in the mother’s body and physiology to allow a new life to begin. During pregnancy, the mother needs to adapt her entire body to support nutrient and oxygen supply, enabling the fetus to grow in the uterus. As a result, these changes not only affect body anatomy but also lead to significant changes at the metabolic level [19,20].

Maternal metabolic adaptation in pregnancy is mediated by the placenta, which is a highly active endocrine organ and most crucial interface between the mother and the fetus. The placenta secretes hormones and growth factors mediating maternal physiological adaptation to pregnancy, resulting in dramatic effects on the mother’s metabolism. Prolactin, human placental lactogen, placental growth hormone and maternal steroid hormones, particularly estrogen and progesterone, are involved [19].

In the early stages of pregnancy, the hormones produced by the placenta increase food intake and promote energy storage, whereas in late pregnancy these reserves are mobilized to support the developing fetus [21,22]. In early pregnancy, prolactin and human placental lactogen lead to pancreatic β-cell mass expansion and decreased apoptosis and, as a result, there is an increase in insulin production [19,22]. Both progesterone and human placental lactogen increase maternal food intake towards the end of the first trimester, during which the metabolic needs of the fetus are still quite low. These events lead to increased adipose tissue mass, which is useful in late pregnancy and breastfeeding [19].

In late pregnancy, however, the placental hormones modulate metabolism, causing a state of insulin resistance to increase glucose concentration in the mother’s blood circulation, thereby enhancing glucose transfer to the fetus, since glucose is the main energy substrate for the fetus, passing across the placenta by simple diffusion [21,23]. Placental growth factor reduces insulin receptor expression and its signaling as well as decreases insulin-stimulated translocation of GLUT4 protein to the plasmatic membrane of skeletal muscle cells. Additionally, progesterone plays a role in insulin resistance development, which decreases insulin’s ability to inhibit gluconeogenesis by the liver and reduces insulin-stimulated glucose uptake by skeletal muscle. Estrogen affects lipid metabolism by increasing HDL levels and improving the lipid profile, while pregnancy hormones overall increase triglycerides and VLDL in maternal blood [19,22].

Moreover, pregnancy is marked by a progressive increase in the level of circulating cortisol; the hypothalamic–pituitary–adrenal axis is altered, and the normal negative feedback loop that suppresses the production of corticotrophin-releasing (CRH) and adrenocorticotropic hormone (ACTH) release in response to high cortisol is reduced. The CRH released by the placenta stimulates cortisol release by the adrenal gland, leading to higher mobilization of nutrients [23,24].

All these mechanisms aim to guarantee the correct growth of the fetus through appropriate glucose intake. Physiological insulin resistance allows for a state of hyperglycemia that allows a higher glucose absorption by the fetus, preventing glucose from being used by maternal tissue [24].

## 3. Overnutrition: Maternal Obesity and GDM

Early pregnancy represents an anabolic state and is associated with increased susceptibility to weight gain; thus, this state, coupled with the harmful metabolic effects of obesity per se, exacerbates the metabolic and molecular alterations observed during obesity. Therefore, obesity and pregnancy represent the perfect storm for causing severe short-term and long-term health consequences to the mother and the fetus [20,25].

The prevalence of overweight and obesity in women of childbearing age is high, and it is a critical cause of short- and long-term problems. For the mother, obesity is a risk factor for GDM: the risk of GDM is 6.5 times higher in pregnancies complicated by obesity, hypertension, and preeclampsia than in normal-weight pregnancies. Moreover, the accumulation of excessive gestational weight also increases the risk of postpartum weight maintenance, stabilizing obesity and promoting obesity-associated cardiometabolic complications, including T2D and CVDs in later life. Offspring also have an increased risk of adverse neonatal outcomes such as preterm birth, being large for gestational age, macrosomia, impaired fetal growth, and hypoglycemia, as well as a long-term risk of developing obesity and metabolic dysfunction [25,26].

In obese pregnant women, normal physiology is disrupted by excess adiposity, which leads to increased insulin resistance, altered lipid metabolism, and inflammation. This disruption increases the nutrient flux from mother to fetus and promotes changes in the placenta. The result is increased nutrient availability, contributing to increased fetal weight with higher adiposity and possible future metabolic outcomes later in life [25,27].

The adipose tissue of obese pregnant women is dysfunctional, shows accelerated insulin resistance, and has decreased expression of PPARγ, all of which are exacerbated by dysregulated secretion of adipokines and circulating inflammation markers [28]. Chronic low-grade inflammation, typical of obese women, activates the immune system in the intrauterine environment, leading to the accumulation of macrophages in the placenta. This, in turn, results in the production of pro-inflammatory cytokines in this organ. As a result, there is uncontrolled placental inflammation that compromises placental function, increasing fatty acids (FAs) in the fetal circulation; furthermore, in mothers with obesity, high glucose levels are exposed as a nuoktrient to the fetus, and this excess of nutrients from maternal glucose stimulates fetal hyperinsulinemia during gestation. Consequently, the gradient of FAs to the fetus through the placenta in obese pregnancies is higher, causing the fetus to gain excessive weight [20,25].

### 3.1. Gestational Diabetes and Its Physiopathology

GDM traditionally refers to abnormal glucose tolerance with onset or first recognition during pregnancy. Nowadays, GDM is the most common pregnancy complication; its prevalence is elevated in Western world areas, partly because of increasing obesity rates and maternal age, leading to side effects in the short and long term [29]. Obstetric and neonatal complications related to GDM are caused by elevated birth weight, which represents a risk factor for the long-term health of the newborn. Recent research demonstrates that in utero exposure to maternal hyperglycemia leads to excessive fetal growth that is already present at the first diagnosis of GDM, which is made through a screening test from the 24th week of gestation. It is also demonstrated that maternal hyperglycemia has a durable negative impact on childhood and adolescent metabolism, rendering GDM one of the main contributors to the spread of intergenerational cardiometabolic diseases [30]. In light of this evidence, early diagnosis of gestational diabetes mellitus (GDM) is crucial, as it predisposes offspring to the early development of type 2 diabetes and cardiovascular diseases and allows for the timely improvement and optimization of clinical management and therapeutic interventions [31,32,33,34]. There are a lot of different approaches to diagnosing GDM; the International Association of the Diabetes and Pregnancy Study Groups and WHO recommend that every pregnant woman between 24th and 28th weeks of gestation undergo the screening test, which is performed using a75 g, 2 h oral glucose tolerance test (OGTT) [31,35,36].

As described above, normal pregnancy is associated with marked changes in glycemic physiology. There is a progressive increase in insulin resistance, predominantly due to increased circulating placental hormones, including growth hormone, corticotrophin-releasing hormone, human placental lactogen, prolactin, estrogen, and progesterone. In early pregnancy, these hormones promote enhanced insulin secretion; in particular, human placental lactogen and growth hormone stimulate maternal pancreatic β-cell proliferation [31,37,38].

Elevated maternal adipose tissue mass during early gestation predisposes women to insulin resistance, culminating in augmented lipolytic activity as pregnancy progresses. The subsequent rise in circulating maternal free fatty acids (FFAs) further intensifies insulin resistance through two mechanisms: impairment of peripheral glucose utilization and upregulation of hepatic gluconeogenesis. Evidence from late-gestation studies demonstrates that insulin sensitivity is reduced by 40–80% in gravidae with normal or elevated body mass index. This heightened insulin-resistant state elevates postprandial glycemia and enhances transplacental glucose flux via facilitated diffusion, thereby increasing substrate availability for fetal anabolism. The temporal pattern of increasing insulin resistance provides the physiological rationale for screening for GDM at 24–28 weeks’ gestation, when metabolic derangement is most pronounced and diagnostic sensitivity is optimized [31,37,38,39,40].

### 3.2. Molecular Biomarkers and Metabolomic Signatures of GDM in the Context of Fetal Programming

GDM is associated with a range of molecular biomarkers, including metabolites, genetic variants, microRNAs (miRNAs), and circulating proteins, which collectively reflect maternal metabolic dysfunction and may influence fetal programming processes. From a developmental origins perspective, the relevance of these biomarkers lies not in their diagnostic value per se, but in their ability to shape the intrauterine environment and induce long-lasting molecular and epigenetic changes in the offspring [41].

Metabolomic studies of GDM have consistently reported alterations in circulating metabolites, particularly branched-chain amino acids (BCAAs). Elevated BCAA levels are associated with insulin resistance, mitochondrial stress, and dysregulated mTOR signaling, pathways that are critically involved in placental nutrient sensing and fetal metabolic programming. Such metabolic disturbances may contribute to altered energy homeostasis and increased susceptibility to obesity and type 2 diabetes later in life [41,42].

Genetic susceptibility to GDM, including single-nucleotide polymorphisms (SNPs) in genes such as *TCF7L2*, *MTNR1B*, and *ADIPOQ*, further modulates maternal glucose metabolism and insulin sensitivity. Although these variants primarily affect maternal physiology, their influence on placental function and fetal nutrient exposure suggests a potential indirect role in shaping offspring metabolic trajectories, particularly when combined with adverse nutritional environments [23,25,26].

Increasing attention has been directed toward circulating miRNAs as mediators linking maternal metabolic status to fetal development. Several miRNAs dysregulated in GDM have been implicated in inflammation, endothelial dysfunction, lipid and glucose metabolism, and insulin signaling. Importantly, placental and circulating miRNAs can modulate gene expression through epigenetic mechanisms and may alter key developmental pathways, including PPAR, mTOR, WNT, and insulin/IGF signaling, thereby influencing fetal growth patterns and long-term disease risk [41,43,44,45].

Proteomic markers such as sex hormone-binding globulin (SHBG), leptin, and adipokines further reflect maternal metabolic health and placental endocrine function [46]. Reduced SHBG levels and altered adipokine profiles observed in GDM and maternal obesity have been linked to impaired placental glucose transport, inflammatory signaling, and epigenetic regulation of genes involved in fetal metabolic programming [47].

Collectively, these biomarker profiles highlight how maternal metabolic disturbances in GDM extend beyond pregnancy outcomes and may act as upstream modulators of fetal epigenetic regulation. By influencing placental signaling pathways, nutrient transport, and gene expression, GDM-associated biomarkers contribute to the intrauterine conditions that predispose the offspring to long-term metabolic and cardiometabolic disorders [48].

### 3.3. Placental Transport Alteration: Biochemical Mechanism

Maternal obesity has been associated with increased placental nutrient transport capacity, although the underlying biochemical mechanisms remain unclear. Evidence from animal models suggests that maternal overnutrition activates key nutrient-sensing pathways regulating placental function [49,50,51,52].

Rosario et al. (2016) showed that a high-fat/high-sugar (HF/HS) maternal diet enhances placental mTOR and insulin/IGF-1 signaling, both of which promote amino acid and nutrient transport [49]. Placentas from HF/HS-fed dams exhibited increased phosphorylation of mTOR targets (rpS6, 4E-BP1) and insulin signaling proteins (IRS-1, Akt), alongside reduced AMPK activation, indicating an overall anabolic placental state linked to fetal overgrowth [49]. Complementary findings by Qiao et al. (2015) demonstrated that a high-fat diet upregulates placental lipoprotein lipase (LPL) and fatty acid transporters (CD36, VLDLr), elevating triglyceride content and fetal free fatty acids [53]. These effects were associated with increased PPARγ and reduced SIRT1 expression—two reciprocal regulators of lipid metabolism—suggesting transcriptional reprogramming of placental lipid handling in maternal obesity. Furthermore, sirtuins (SIRT1 and SIRT3) play key roles in fetal programming by controlling oxidative metabolism, autophagy, and mitochondrial function. Obesity-induced suppression of sirtuin activity contributes to oxidative stress, impaired mitochondrial integrity, and potentially transgenerational metabolic dysfunction [54]. Collectively, maternal obesity appears to overstimulate placental nutrient-sensing networks (mTOR, insulin/IGF-1, PPARγ) while inhibiting energy homeostasis pathways (AMPK, SIRT1), leading to excessive nutrient transfer and long-term metabolic consequences for the offspring [49,53,55,56,57,58].

## 4. Long-Term Outcomes: Metabolic Consequences

### 4.1. GDM

In addition to obstetric and neonatal complications, GDM is associated with adverse long-term health effects on offspring. There is some evidence, albeit uncertain, correlating GDM with an increased obesity risk in offspring. Zhao P. et al. demonstrated the association between GDM and childhood obesity in 12 different low- and high-income countries; a total of 4740 children aged between 9 and 11 years participated in the study. The results of this study demonstrate that children of mothers whose pregnancies were complicated by GDM show increased BMI, central adiposity, and body fat percentage compared with children of mothers whose pregnancies were not complicated by GDM. However, the results varied after adjustment for maternal BMI, suggesting that maternal weight may have a stronger impact than GDM on the long-term risk of obesity in offspring [59].

The Hyperglycemia and Adverse Pregnancy Outcome Follow-up Study (HAPO-FUS) prospectively evaluated 4832 offspring aged 10–14 years born to mothers enrolled in the original HAPO cohort. Findings revealed persistent metabolic consequences of maternal hyperglycemia on long-term offspring glucose homeostasis, notably extending to glycemic levels below current GDM diagnostic thresholds. A dose-dependent relationship emerged between maternal antenatal glycemia and offspring metabolic parameters across the continuum of glucose concentrations. Maternal glycemic dysregulation correlated with increased prevalence of impaired fasting glucose, glucose intolerance, elevated glycated hemoglobin, and pediatric obesity in adolescent offspring. Conversely, maternal glucose elevation demonstrated inverse associations with offspring insulin sensitivity and homeostatic model assessment (HOMA) indices at 14 years, with these relationships persisting after statistical adjustment for maternal body mass index, offspring BMI, and familial diabetes predisposition. Furthermore, recent evidence shows that there is increased glucose-linked hypothalamic activation in offspring who were previously exposed to maternal obesity and GDM in utero, which represents one possible mechanism for this increased childhood obesity risk [31,32,60,61].

Recent epidemiological studies show a relationship between an increased risk of cardiometabolic diseases in adulthood for offspring of women whose pregnancy has been complicated by GDM. A large study conducted by Yu Y. et al. demonstrated an association between gestational diabetes and an increased rate of early-onset cardiovascular diseases (CVD; ≤40 years old) in offspring. This study included 2,432,000 subjects who were born between 1977 and 2016, evaluating how in utero exposure to gestational diabetes and maternal type II or type I diabetes influenced the predisposition to cardiovascular diseases in adulthood. Based on hospital data, they discovered a strong association between in utero exposure to maternal diabetes and early onset of cardiovascular diseases such as hypertension, heart failure, deep vein thrombosis and pulmonary embolism. The incidence ratio of offspring exposed to every type of maternal diabetes was increased by 29% compared to non-exposed offspring, and the increase in risk was observed in every age group (<20 years old; >20 years old) [33,62,63,64,65].

Surprisingly, both type I and type II diabetes during pregnancy increased the onset risk of diseases in offspring. Several studies demonstrate that perinatal exposure to abnormal glucose concentrations in the environment increases the risk that offspring develop obesity, type II diabetes and associated diseases. In 2011, Steculorum et al. designed a study that used a mouse model of maternal insulin deficiency induced by STZ (a pancreatic β-cell toxin) injections during pregnancy to study the effects of maternal hyperglycemia on offspring [66]. Compared with control pups, STZ-derived pups had heavier body weights at birth, and this elevated body weight persisted into adulthood and was also associated with an increase in food intake during adulthood, suggesting that in utero exposure to hyperglycemia affects neuronal circuits involved in the regulation of hunger and satiety. Blood glucose and insulin levels were higher in adult animals born to diabetic mothers compared with control mice; furthermore, serum leptin levels were doubled compared to control subjects, and WAT histological analysis showed adipocyte hyperplasia, suggesting possible leptin resistance induced by in utero exposure to maternal hyperglycemia. Recently, compromised development of hypothalamic circuits due to maternal hyperglycemia and impaired development and axonal growth of ARH neurons has been demonstrated [66,67,68,69,70]. Recently, it has been highlighted that maternal obesity impacts fetal brain volume [71].

### 4.2. Obesity

Obesity, in addition to increasing the risk of adverse pregnancy outcomes, mainly represented by obstetric complications caused by excessive fetal growth, also has the capacity to predispose newborns to an increased risk of obesity and cardiometabolic diseases in adulthood, such as hypertension, type II diabetes, and dyslipidemia [3,72]. It is well established that maternal obesity is associated with adverse outcomes for offspring long-term health. Maternal obesity can have an adverse impact on newborn body composition and adiposity, which are related to adverse cardiometabolic outcomes, higher cancer morbidity and an increased mortality ratio in general. The cohort study conducted by Eriksson et al., including 2003 subjects born in Helsinki, the capital of Finland, between 1934 and 1944, focused on the analysis of physical characteristics (BMI, body composition and circumferences), clinical characteristics and cardiometabolic risk factors, attempting to discover the association between maternal BMI and subject health; at the time of the study, the subjects were aged 62 years on average [73]. Regarding anthropometry, they assessed height, weight, BMI, abdominal circumference and body composition through bioimpedance analysis. Moreover, clinical parameters, including blood pressure and glucose tolerance, were evaluated. The results show that there is a positive relationship between maternal BMI and offspring BMI, and it is also positively correlated with offspring body fat percentage. There is a relationship between maternal BMI and birth weight as well as body fat percentage in adulthood; in fact, despite high weight at birth, the children of mothers with a low BMI show, during adulthood, a lower percentage of body fat compared to children of mothers with a high BMI. Increased adiposity in offspring can explain the relationship between high maternal BMI and increased risk of cardiometabolic diseases [73,74,75].

The relationship between maternal obesity and increased mortality rates for cardiovascular events, such as myocardial infarction, stroke, and peripheral artery disease, in offspring is well established [74].

For example, Reynolds et al. conducted a study on 37,709 Scottish subjects aged between 34 and 61 years, based on hospital data that certified death or hospitalization for cardiovascular events, to assess how maternal BMI (calculated using height and weight data collected during the prenatal period) could affect the risk. The results showed an increase in mortality in offspring of overweight or obese mothers, particularly for cardiovascular events; moreover, approximately 7.6% of offspring were hospitalized for a cardiovascular event. In conclusion, there is a positive relationship between maternal BMI and offspring cardiovascular risk [76,77,78].

The negative effect of maternal obesity on offspring health might be mediated by an alteration in the inflammatory state and an increase in oxidative stress due to excess adipose tissue. Oxidative stress and inflammation associated with obesity are well known as triggers for insulin resistance and other metabolic alterations, such as modifications in lipid homeostasis, that can lead to the development of type II diabetes, non-alcoholic fatty liver disease (NAFLD), and cardiovascular diseases. Increased oxidative stress and inflammation markers have been found in the placenta of obese mothers, suggesting a decisive role of an abnormal intrauterine environment in modulating fetal programming [76,79].

Pregnancy is normally an inflammatory state caused by the remodeling of immunological system to allow embryo implantation, growth and life, regulated by a delicate balance of pro-inflammatory and anti-inflammatory cytokines; in pregnancies complicated by obesity, this balance is shifted mainly towards a pro-inflammatory state. Therefore, fetuses of obese mothers are exposed to an imbalance of cytokines, as well as nutrients that can negatively affect fetal programming, leading to adverse long-term effects in the offspring. Evidence of a key role of inflammation in fetal programming was provided by Reynolds et al. in 2015, thanks to a study where supplementation with an anti-inflammatory molecule, the conjugated linoleic acid, led to a reduction in the expression of pro-inflammatory cytokines such as TNF-α and IL-1β and metabolic dysfunction in offspring [76,79].

In 2014, Alfaradhi et al. analyzed the role of oxidative stress and altered fat metabolism, typical of pregnancies complicated by obesity, in the pathogenesis of NAFLD in offspring. This study was conducted on a mouse model; female mice were fed an obesogenic diet, and the offspring were analyzed when 8 weeks old (after weaning and fed with a normocaloric diet) before significant differences in weight and adiposity were found compared to the offspring of mothers fed a control diet. The results of this study highlight how maternal obesity led to profound changes in the offspring’s liver histology and physiology; histological analysis shows an increase in hepatic lipid content compared to control subjects. Plasma analysis showed a decrease in HDL cholesterol, an increase in fasting insulin, TNF-α and DNA oxidative damage markers and hepatic protein oxidation. Also, variations in protein expression involved in oxidative stress homeostasis were found, including reduced expression of NADPH oxidase and glutathione peroxidase, an increase in catalase and a decrease in protein levels involved in lipogenesis and lipolysis; in particular, an increase in PPARγ expression was discovered. Regarding mitochondrial damage, there was an increase in the activity of complexes I and II and a significant reduction in cytochrome C expression [80,81].

The defining organ of obesity is adipose tissue; understanding the development and biology of this tissue is crucial for understanding the pathogenesis of obesity and associated diseases. Adipose tissue develops prenatally, with marked expansion during the third trimester of pregnancy; at birth, fat mass accounts for approximately 15% of neonatal body weight, and sexually dimorphic differences in body composition are already present. It is evident that the distribution of adipose tissue depends on sex and environmental factors, such as exposure to maternal obesity in utero, which can modify its programming as it does for all other tissues [80,81].

**Figure 2:**
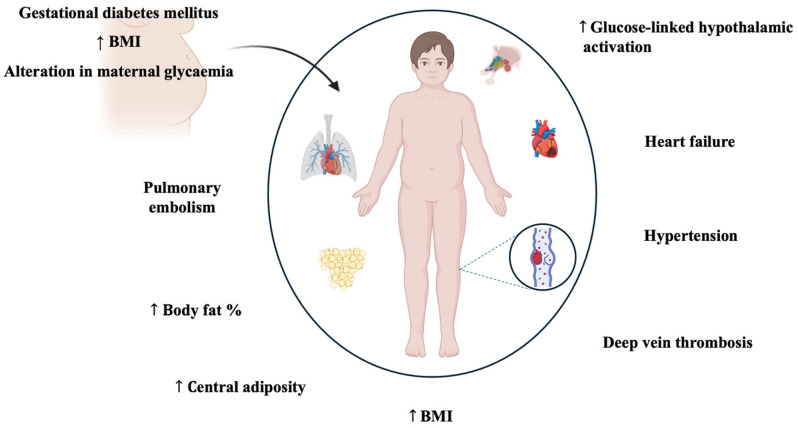
Long-term outcomes: metabolic consequences. This figure illustrates the long-term metabolic consequences in offspring associated with adverse prenatal and early-life exposures. These metabolic outcomes are long-term effects that emerge and persist during growth and development, extending from childhood into young adulthood (as indicated by the arrow from the womb to the young adult). Metabolic outcomes include an increased risk of obesity, insulin resistance, type 2 diabetes, and cardiovascular diseases. Original figure created by the authors using Microsoft PowerPoint Office 365.

Savva C. et al. investigated how prenatal exposure to maternal obesity can modulate offspring adipose tissue programming in a sex-dependent way [82]. The study was conducted on mouse models assessing the percentage and distribution of white adipose tissue (WAT), both subcutaneous (SAT) and visceral (VAT), in offspring using nuclear magnetic resonance and metabolic profiling. Mice dams were fed either a control diet (CD) or a high-fat diet (HFD) for 6 weeks; after weaning, all offspring, both from mothers fed the HFD diet (mo-HFD) and mothers fed the control diet (mo-CD), were fed an HFD. At the age of 5 weeks, offspring body weight was sex- and maternal diet-dependent; offspring of mo-HFD mothers increased in weight compared to offspring of mo-CD, and this increase was more evident in male offspring (M-moHF) compared to female offspring (F-moHF). Female offspring showed a higher percentage of body fat compared to M-moHF. Histological analysis of adipose tissue showed that in M-moHF VAT there is hyperplasia and a decrease in hypertrophy of adipocytes compared to M-moC and F-moHF; instead, in SAT, adipocytes are hypertrophic and fewer in number compared to F-moHF. To assess metabolic state, fasting glucose levels were measured, as well as insulin levels and fatty acids profiles of VAT and SAT. Analysis showed a predictable increase in glycemia in mo-HF offspring regardless of sex, compared to mo-C offspring; this increase was more evident in M-moHF. This study highlights how maternal obesity leads to negative long-term outcomes in metabolism and functionality of adipose tissue more than post-natal exposure to an obesogenic diet, exacerbating the negative effects of excessive fat storage and dysfunction of adipose tissue. Moreover, it has been noted that female subjects seem to have greater protection against the adverse effects of lipo-toxicity caused by dysfunctional adipose tissue, probably through a differentiated expression of sex-dependent genes involved in adipogenesis (Figure 2) [58,82,83].

We acknowledge that the literature shows heterogeneous findings regarding the association between gestational diabetes mellitus (GDM) and offspring obesity, particularly after adjustment for maternal pre-pregnancy BMI. Several recent cohort studies indicate that the association between GDM and increased BMI or risk of overweight in early childhood is attenuated or even null after adjustment for maternal pre-pregnancy BMI, indicating potential confounding effects attributable to maternal adiposity [84]. Conversely, other contemporary research demonstrates independent and joint effects of maternal GDM and overweight on offspring adiposity trajectories from birth into early adolescence, with maternal GDM maintaining a positive association even when accounting for BMI and other covariates [85]. These divergent findings may reflect differences in outcome definitions (e.g., BMI z-score vs. skinfold thickness), timing of follow-up, population characteristics (e.g., genetic background, diet, postnatal environment), and methods of covariate adjustment. Additionally, maternal BMI itself may act both as a confounder and as a mediator in the causal pathway, further complicating interpretation. Taken together, the evidence suggests that maternal metabolic status—including GDM and overweight/obesity—contributes to offspring adiposity risk through complex and potentially interacting biological and environmental mechanisms.

## 5. Critical Micronutrient Imbalance: Implications for Developmental Programming

The importance of addressing micronutrient deficiencies in this review stems from their critical role in shaping fetal development and long-term health outcomes. While much focus has been placed on maternal overnutrition and gestational diabetes mellitus, inadequate intake of essential micronutrients—including iron, folate, iodine, vitamin A, and vitamin D—can profoundly affect cellular processes such as DNA synthesis, epigenetic regulation, and organogenesis [86,87,88]. These deficiencies contribute not only to immediate pregnancy complications but also to persistent alterations in fetal growth, neurodevelopment, and metabolic programming, often mediated through epigenetic mechanisms [89,90]. Thus, understanding micronutrient status is essential for a comprehensive view of how maternal nutrition influences offspring health across the DOHaD continuum.

### 5.1. Iron

During the second half of gestation, a physiological increase in erythropoiesis occurs to support the oxygenation of the fetus. The erythrocyte mass increases by 10–15%, but this increase is concealed by the dilution effect of plasma volume, which increases by 40–50% compared to the beginning of pregnancy. The massive expansion of plasma volume involves a dilution of erythrocyte mass and hence produces anemia, which is a physiological adjustment, to allow efficient perfusion of oxygen to the fetus [90,91].

To support increased erythropoiesis, maternal daily iron intake should increase from 18 to 27 mg/day. Often, a normal diet cannot satisfy the iron requirement, causing pathological iron deficiency anemia [92].

Iron deficiency during pregnancy leads to short- and long- term complications for the newborn, such as fetal growth restriction and low birth weight, increased risk of premature birth, predisposition to anemia in the first month of life, impairment of the immune system, deficits in cognitive and behavioral development, reduction in IQ (intelligent quotient), neurobehavioral disorders such as ADHD (attention deficit hyperactivity disorder) or ASD (autism spectrum disorder) or mood disorder and emotional dysregulation [93,94,95].

Iron is very important for neuronal development and is essential for the myelination of nerve fibers, mitochondrial function, and hippocampus development. Its deficiency has a negative effect on learning and memory, neurotransmitter synthesis such as dopamine, serotonin, norepinephrine, Gamma-aminobutyric acid (GABA), and acts as an enzyme co-factor [96,97]. The persistence of anemia in the first month of life also affects synapsis development, causing permanent learning and memory deficits [94,95,98,99].

Both iron deficiency and iron overload during pregnancy pose significant risks to maternal and fetal health [100]. Iron excess in pregnancy typically occurs following iron supplementation in non-anemic women, where iron overload can lead to preeclampsia and GDM. Specifically, Park et al. identified placental ferroptosis as a key mechanism linking iron overload to severe complications such as preeclampsia and GDM [101]. Excessive iron damages trophoblast cells, triggering oxidative stress and the release of toxic molecules into the maternal circulation, thereby compromising maternal vascular function—a hallmark of preeclampsia—and impairing fetal development. Furthermore, research indicates that high body iron stores in early pregnancy are also associated with an increased risk of developing GDM [102]. Consequently, routine iron supplementation during pregnancy should be avoided in favor of a personalized approach based on individual hemoglobin levels to mitigate the risks of fetal growth restriction and gestational hypertension [103,104].

Placental iron transport is essential for fetal growth, with transferrin receptor 2 (TfR2) playing a pivotal role in maintaining iron homeostasis. Significant differences in placental transferrin receptor levels have been observed in pregnant women with high GWG, which may be linked to variations in fetal iron uptake. A study by Ege et al. (2025) demonstrated that excessive weight gain leads to the overexpression of TfR2, resulting in excessive iron absorption and subsequent iron overload within trophoblast cells [105]. This is accompanied by an increase in lipid peroxidation—a hallmark of ferroptosis—which can trigger oxidative damage, inflammation, and placental dysfunction, potentially leading to preeclampsia and GDM. Furthermore, ferroptosis-induced placental dysfunction may compromise the transport of nutrients and oxygen, resulting in adverse fetal outcomes. Given the critical role of iron homeostasis during pregnancy, careful monitoring of maternal weight gain and iron metabolism is essential [105,106].

Finally, an association between higher early-pregnancy ferritin concentrations and lower cord blood DNA methylation at three CpGs was found to be long-lasting, remaining partially evident in older children [107,108].

In summary, iron plays a pivotal role in supporting placental function, fetal growth, and neurodevelopment, with long-lasting consequences and implications extending from early development to adult health [92,93]. Both deficiency and excess disrupt iron homeostasis, leading to altered erythropoiesis, placental dysfunction, oxidative stress, ferroptosis, and epigenetic modifications that can program offspring susceptibility to neurodevelopmental disorders and metabolic disease [99,100,101,108]. While iron requirements increase during pregnancy (recommended intake: 27 mg/day), indiscriminate supplementation may be harmful [92,103,104]. Therefore, individualized assessment of iron status is essential to optimize maternal-fetal outcomes and safeguard the long-term health of future generations [100,101,102].

### 5.2. Folic Acid (Vitamin B9)

Folic acid is a micronutrient necessary for human survival and is involved in essential cellular events: synthesis of DNA and cell proliferation, erythrocyte formation, the vitamin B12-dependent remethylation of homocysteine to methionine, DNA and histone methylation. Folic acid requirement in adults is approximately 200 μg/day; in pregnant women, it doubles to 400 μg/day by the third trimester due to the formation of new fetal and maternal tissue [109].

Folic acid deficiency inhibits DNA synthesis due to reduced purine basis, causing cell cycle arrest in the S phase and resulting in megaloblastic-type red blood cells, with unusually large dimensions (macrocytes), fragile membranes, altered nucleus shapes and delayed maturation. These changes are called megaloblastic anemia. Low folic acid levels lead to the incorporation of uracil into DNA in place of thymine, resulting in double-strand breaks as well as chromosome instability, which can lead to apoptosis [110]. Inadequate intake of folic acid produces hyperhomocysteinemia, which is a risk factor for cardiovascular diseases and diseases associated with cognitive deficiency. An excessive amount of homocysteine affects the endothelial function of blood vessels because of oxidative stress, smooth muscle cell proliferation, higher platelet aggregation, decreased fibrinolysis, LDL oxidation and placental ischemia [110]. Inadequate levels of folic acid in early pregnancy increase the risk of congenital deformities such as neural tube defects (NTDs) and spina bifida due to alterations in cell division and gene expression.

Fetal deformities due to inadequate intake of folic acid during pregnancy cause structural defects that affect the central nervous system [111,112].

In relation to birth defects, AdoMet (S-adenosylmethionine) and its role in transmethylation reactions have received attention. AdoMet is used in the methylation of phospholipids, proteins, DNA, RNA, amino acids, neurotransmitters and a few other molecules. Variation in the methylation of critical CpG loci in DNA can modulate gene expression and cellular differentiation. Moreover, altered methylation activity may interfere with normal fetal growth and development in different ways; for example, hypomethylation can occur when AdoMet levels are very low. In fact, women with low methionine intake show an increased risk of having a baby with an NTD [110].

Emerging data from contemporary investigations demonstrate an association between inadequate maternal folate status during gestation and aberrant neurodevelopmental trajectories in offspring, with implications extending to long-term neuropsychiatric outcomes. Offspring exposed to suboptimal intrauterine folate environments exhibit distinctive neuroanatomical alterations, including diminished cerebral white matter and subcortical gray matter volumes, increased cortical thickness in left frontal regions, and reduced surface area bilaterally across frontal and temporal cortices. These findings suggest that insufficient prenatal folate availability adversely impacts fetal craniofacial development independent of NTD occurrence, potentially through mechanisms involving impaired neural crest stem cell proliferation. This developmental disruption may result in compromised intracranial volume, thereby imposing spatial constraints on normal brain expansion and maturation [113].

Folic acid deficiency is also very important to the newborn child’s metabolism. A nine-year prospective study carried out in Boston involving 1517 mother–son couples of different ethnicities has shown how folic acid deficiency was involved in a transgenerational transition of obesity and how its supplementation improved the offspring’s metabolic parameters. Indicators of metabolic health included BMI, plasma insulin, leptin, and the leptin/adiponectin relation. Respectively, 29% and 25% of women were overweight or obese before pregnancy and showed lower folate concentration compared to normal-weight women; 38% of children were overweight at the age of 2–9 years, indicating that maternal folic acid concentration is inversely proportional to the Z-score (number of standard deviation) of the BMI of the baby. In particular, it was shown that there was a strong increase in the Z-score of BMI for children whose mothers had a plasma concentration of folate below the 25th percentile; such an association was even stronger when mothers were overweight or obese. The children of overweight or obese mothers with adequate folate plasma concentrations showed a 43% reduction in obesity risk. Low maternal plasma folate is linked to insulin and leptin concentration and to a reduction in the leptin/adiponectin ratio. The children of obese or overweight mothers with folate deficiency showed increased Z-scores of insulin (0.39 units) and similarly for leptin. The leptin/adiponectin ratio was reduced by 0.43 units. Children of obese or overweight mothers but with adequate plasma folate concentration showed smaller fluctuations in Z-scores for all the parameters [114].

Finally, the body effectively manages folate levels through urinary excretion; thus, its water-soluble nature ensures that the nutrient remains non-toxic even at high intakes [115,116].

In summary, folic acid is a key regulator of DNA synthesis, methylation processes, and epigenetic programming during early development, making it central to fetal organogenesis and long-term metabolic and neurocognitive health [109,110]. Adequate maternal folate status protects against neural tube defects and supports proper brain development, while deficiency is associated with persistent alterations in offspring neurodevelopment and an increased risk of obesity and insulin resistance later in life [112,113,114]. The recommended intake during pregnancy is 400 μg/day, ideally initiated preconception, highlighting folate’s critical role in shaping health trajectories across generations [109,115].

### 5.3. Iodine

Iodine is a micronutrient essential for thyroid hormone synthesis and for normal fetal development [117,118]. The fetal thyroid does not mature until mid-gestation, so the fetus depends on maternal thyroid hormones, which cross the placenta during early pregnancy. Maternal hypothyroidism caused by iodine deficiency is associated with adverse pregnancy outcomes and impaired fetal growth, such as spontaneous abortion, premature birth, fetal death, low birth weight, neonatal mortality, growth restriction, altered cognitive development (cretinism, language disorders, altered motor development) [119].

During pregnancy, iodine requirements increase because of the rising production of thyroid hormones, urinary excretion loss and the fetus’s need for fetal thyroid hormones synthesis. The World Health Organization (WHO) recommends that pregnant women take 250 μg of iodine per day during pregnancy compared to 150 μg/day for adults [119,120].

The first phase of fetal neurological development starts in the second half of the first trimester and depends on maternal thyroid hormones since the fetal thyroid does not mature until 18–20 weeks of gestation. This developmental period encompasses neural progenitor cell expansion and the initiation of neuronal translocation within the cerebral cortex, hippocampal formation, and medial ganglionic eminence [120].

Fetal thyroid activity starts at the beginning of the second trimester; however, the full development of the pituitary-portal vascular system in the fetus does not occur until 18–20 weeks of gestation.

The second phase of thyroid hormone-dependent neurodevelopment encompasses neural progenitor proliferation, cellular translocation, axonal extension, dendritic arborization, synaptic formation, glial lineage specification and migration, along with the initiation of myelin sheath deposition [121].

The most serious consequence of iodine deficiency during pregnancy is cretinism, marked by a profound intellectual developmental disorder. Cretinism is always associated with significant impairment in mental function, such as hypothyroidism and/or defects in hearing, speech, stance, gait, and growth. Cretinism manifests in two distinct clinical phenotypes: neurological cretinism, the predominant variant, presents with intellectual disability, sensorineural deafness with mutism, strabismus, spastic diplegia, and abnormalities in posture and ambulation; conversely, myxoedematous (hypothyroid) cretinism, the less prevalent form, exhibits attenuated cognitive impairment, growth retardation, thyroid hypofunction, alongside diverse somatic manifestations including dry and coarse skin texture, hoarse vocalization, and delayed pubertal development [122,123,124,125].

Pregnant women are susceptible to iodine toxicity arising from various sources, including potable water, dietary intake, prenatal supplements, and iodine-containing medications. Overexposure to iodine during pregnancy is associated with a higher risk of birth complications and imbalances in maternal glucose and cholesterol levels [126]. Moreover, exposure to supraoptimal iodine levels during gestation and breastfeeding triggers adult-onset hypothyroidism in rat offspring. This condition is driven by epigenetic silencing—specifically, DNA hypermethylation and histone remodeling—of essential thyroid-regulating genes such as Tshr, Nis, Tpo, and Pax8. Consequently, the offspring exhibit structural thyroid alterations, elevated oxidative stress, and disrupted hormonal profiles, indicating a permanent developmental programming toward endocrine dysfunction [127].

In summary, iodine is indispensable for thyroid hormone synthesis, which is crucial for fetal brain development, particularly during early gestation when the fetus relies entirely on maternal thyroid hormones [119,121]. Both iodine deficiency and excess can result in long-term neurodevelopmental impairments and endocrine dysfunction through disrupted thyroid signaling and epigenetic mechanisms [119,126,127]. To ensure optimal neurodevelopment and prevent irreversible cognitive deficits, the World Health Organization recommends an iodine intake of 250 μg/day during pregnancy, emphasizing the importance of maintaining iodine balance for lifelong offspring health [119].

### 5.4. Vitamin A

Vitamin A is a vital micronutrient during pregnancy for both the mother and the baby. It plays a key role in vision, as it is a precursor of rhodopsin, a crucial pigment in retinal photoreceptor cells. It is also involved in cell differentiation, helps preserve eye health, and prevents xerophthalmia; its deficiency remains the leading cause of avoidable blindness worldwide. In addition, vitamin A contributes to bone formation, protects the skin and mucous membranes, supports the development and maintenance of epithelial tissues, is important for the proper functioning of reproductive organs, strengthens the immune system, and is essential for normal embryonic development [128,129,130].

During pregnancy, vitamin A requirements rise by approximately 10–20%, and the recommended intake for pregnant women is 800 µg/day, with particular importance in the third trimester due to the rapid growth and development of the fetus. The World Health Organization defines vitamin A deficiency (VAD) in pregnancy as serum retinol levels below 0.70 µmol/L. However, the reliability of serum retinol as an indicator of vitamin A status during pregnancy—especially in the final trimester—and in the presence of inflammation has been questioned. Physiological changes in pregnancy modify the relationship between liver vitamin A stores and circulating retinol levels. In addition, retinol-binding protein (RBP), which transports retinol in the blood, is an acute-phase protein whose concentration can fluctuate during inflammatory conditions. Since pregnancy itself is characterized by a state of physiological inflammation due to the immune adaptations required to support fetal survival, serum retinol levels and their standard cut-off values may underestimate true vitamin A status in late pregnancy [128,129].

The primary organs and systems affected by vitamin A deficiency (VAD) include the heart, central nervous system and its associated structures, the circulatory, urogenital, and respiratory systems, as well as the skull, skeleton, and eyes. Vitamin A is essential for the early formation of the primitive heart and circulatory system and for the proper development of the rhombencephalon; deficiency during this critical developmental period can cause serious malformations, including early embryonic death. VAD can also result in impaired iron mobilization, disrupted cellular differentiation, weakened immune function, growth delay, and xerophthalmia. The term xerophthalmia refers to eye disorders caused by VAD and includes night blindness, which is often one of the earliest clinical signs of this micronutrient deficiency [131].

Vitamin A deficiency is a major cause of anemia. Recent studies have shown that vitamin A supplementation during pregnancy increases hemoglobin levels. Although the exact mechanism linking serum retinol levels and anemia is not yet fully understood, it is clear that vitamin A influences hematopoiesis, iron metabolism, and immune function. Regarding the immune system, observational studies in Sub-Saharan Africa have associated vitamin A status in HIV-infected pregnant women with higher rates of vertical (mother-to-child) transmission of the infection and increased infant mortality [128,129].

Moreover, several studies show a connection between maternal vitamin A deficiency and diabetes mellitus in adulthood and gestational diabetes. During pregnancy, as shown by several animal studies, vitamin A deficiency affects fetal development negatively, particularly in endocrine pancreas development, suggesting a dominant impact of this micronutrient in the diabetic pathogenesis [128,129].

It is widely known that, conversely, excess vitamin A has teratogenic effects. A vitamin A intake exceeding 10,000 UI at the beginning of the first trimester can lead to serious congenital malformations involving the central nervous system and cardiovascular system, causing miscarriage in the most serious cases. The mechanism of action by which vitamin A exerts teratogenicity is attributed to the influence of high concentrations of certain retinoic acid metabolites (such as trans-retinoic acid and 13-cis-retinoic acid) on the function of genes during critical periods of organogenesis and embryogenesis [128,129].

However, the evidence highlights the necessity of cautious vitamin A intake during pregnancy; even if non-lethal or non-teratogenic, excessive levels may impair the offspring’s future metabolism [132] and are associated with higher GDM risks [133].

In summary, vitamin A is essential for embryogenesis, organ differentiation, immune competence, and visual function, exerting profound effects on fetal development and long-term metabolic programming [128]. Deficiency during pregnancy is associated with congenital anomalies, impaired immune function, altered pancreatic development, and increased susceptibility to metabolic disease in later life, whereas excess intake carries well-established teratogenic risks [128,132,133]. The recommended intake for pregnant women is approximately 800 μg/day, underscoring the need for carefully regulated vitamin A status to protect both immediate pregnancy outcomes and future generational health [128].

### 5.5. Vitamin D

Vitamin D is a micronutrient essential for human health and is mainly involved in calcium metabolism, particularly responsible for calcium and phosphate homeostasis by acting on the kidney, intestine and bone tissue. Vitamin D action expresses itself through a hormone-like mechanism. The receptors of vitamin D (*VDR*) are cytosolic, and once the *D-VDR* complex is formed, it moves into the nucleus, where it associates with the RXR factor, thereby mediating gene transcription [134]. *VDR* can be found in a multitude of tissues and performs the following actions: it regulates the immune system, particularly strengthening innate immunity and suppressing adaptive immunity, decreases infection risk, protects against autoimmune diseases, reduces respiratory infections, reduces cardiovascular diseases and hypertension, prevents cancer through antiproliferative and antineoplastic functions, and stimulates insulin production from pancreatic β-cells. In recent years, the relationship between vitamin D deficiency and negative pregnancy outcomes has been widely studied, leading to the hypothesis that low vitamin D levels may be linked to a higher risk of preeclampsia, as suggested by observational studies, as well as gestational diabetes [135,136].

GDM is defined as glucose intolerance that is first identified during pregnancy. Vitamin D may influence glucose metabolism through multiple biological mechanisms, including insulin secretion and insulin sensitivity. Current evidence suggests that vitamin D deficiency may act as a contributing factor that increases the risk of developing gestational diabetes. However, because vitamin D deficiency is more prevalent among obese women—who already have a higher incidence of gestational diabetes—it is difficult to clearly determine a direct causal relationship between vitamin D status and GDM [135,137,138,139].

It is now widely recognized that vitamin D deficiency during pregnancy is linked to adverse outcomes not only at birth but also for long-term health of both mothers and their children. Low vitamin D levels in pregnant women, especially those who are obese, have been associated with disruptions in glucose and lipid metabolism, which may contribute to higher risks of diabetes and obesity later in life for their offspring. Observational studies have found that obese pregnant women tend to exhibit lower circulating vitamin D levels compared with non-obese women, likely due to greater sequestration of vitamin D in adipose tissue [140,141,142].

A number of cohort and observational studies have also reported associations between low maternal vitamin D status and variations in neonatal body measurements, including increased risk of being overweight or higher adiposity in infancy and early childhood. For example, maternal vitamin D deficiency has been linked to greater odds of overweight in infants at one year of age and higher body mass index (BMI) and waist circumference in preschool-aged children [143,144].

Additionally, lower prenatal vitamin D levels have been associated with differences in offspring fat mass, with some evidence suggesting lower fat mass at birth but increased adiposity by 6 years of age, independent of maternal obesity [140,145].

In gestational diabetes, women with GDM often show lower circulating vitamin D levels, along with increased expression of vitamin D receptor (VDR) and peroxisome proliferator-activated receptor gamma (PPAR-γ) mRNA in adipose tissue. These changes may influence the regulation of adipocyte development and fat storage pathways, potentially contributing to the progression of adipogenesis in GDM [140,146].

Several observational studies have also found that insufficient vitamin D status is associated with a higher risk of insulin resistance and the development of GDM, suggesting that low serum 25-hydroxyvitamin D may be an independent risk factor for impaired glucose metabolism during pregnancy [147,148].

Emerging clinical evidence indicates that supplementation with vitamin D can improve insulin sensitivity and glucose tolerance in women with GDM, likely by modulating insulin signaling pathways via the VDR and reducing inflammation [149].

Furthermore, it is hypothesized that maternal vitamin D deficiency may have long-term effects on the health of the offspring. Preclinical studies have shown that inadequate maternal vitamin D can lead to insulin resistance and metabolic alterations in the offspring, potentially through inflammatory and epigenetic mechanisms [140,150].

Regarding epigenetic mechanisms, vitamin D, through its interaction with the vitamin D receptor (VDR), can modulate the activity of chromatin-modifying enzymes, including DNA methyltransferases (DNMTs), histone acetyltransferases (HATs), and histone deacetylases (HDACs). Experimental studies in different animal models have demonstrated that vitamin D can influence DNA methylation patterns and induce histone modifications such as methylation and acetylation. In addition, vitamin D can regulate microRNA (miRNA) expression, leading to reduced expression of inflammation-related miRNAs, thereby exerting anti-inflammatory and regulatory effects at the epigenetic level [134,151,152,153].

Finally, it is important to emphasize that maternal vitamin D deficiency can predispose offspring to obesity by promoting inflammation, oxidative stress, abnormal fat development (adipogenesis), altered adipokine signaling, and dysbiotic gut microbiota, with potential epigenetic changes; supplementing deficient mothers during pregnancy may mitigate these metabolic risks in their children [140].

In summary, vitamin D acts as a pleiotropic hormone influencing calcium metabolism, immune regulation, glucose homeostasis, and epigenetic modulation of gene expression. Maternal vitamin D deficiency has been linked to adverse pregnancy outcomes, increased risk of gestational diabetes, and long-term metabolic consequences for offspring, including obesity and insulin resistance. Adequate maternal vitamin D status supports healthy fetal growth and may reduce chronic disease risk later in life. Current recommendations generally suggest an intake of 600 IU (15 μg)/day during pregnancy, with higher doses often required in deficient populations to ensure optimal maternal–fetal health [135,140].

### 5.6. Nutrient–Nutrient Interactions

Maternal micronutrient status is not determined solely by individual nutrient intake, but also by complex nutrient–nutrient interactions that influence absorption, bioavailability, metabolism, and biological efficacy. For example, vitamin C enhances non-heme iron absorption by reducing ferric to ferrous iron, thereby improving iron bioavailability and reducing the risk of iron-deficiency anemia [154]. Conversely, excessive iron may interfere with the metabolism of other micronutrients and promote oxidative stress. Similarly, vitamin D and calcium are tightly interdependent, as vitamin D is essential for intestinal calcium absorption and skeletal mineralization; inadequate vitamin D status can impair calcium utilization despite sufficient intake [134]. These interactions are particularly relevant during pregnancy, when micronutrient requirements are increased and imbalances may exacerbate placental dysfunction, disrupt fetal development, and contribute to long-term metabolic and epigenetic programming of offspring. Therefore, considering nutrient–nutrient interactions is crucial for developing effective, personalized nutritional strategies aimed at optimizing maternal and intergenerational health. Folate and vitamin B12 act synergistically in one-carbon metabolism, and deficiency of vitamin B12 can impair folate-dependent DNA synthesis and methylation, potentially masking megaloblastic anemia while exacerbating epigenetic dysregulation during fetal development [110,155]. Iodine and selenium are functionally linked in thyroid hormone metabolism, as selenium-dependent deiodinase enzymes regulate thyroid hormone activation and inactivation; inadequate selenium status may therefore amplify the adverse neurodevelopmental effects of iodine deficiency during pregnancy [119,156].

## 6. Epigenetic Mechanisms and Transgenerational Transmission

Environmental and lifestyle factors profoundly influence human development, particularly during critical windows such as conception, gestation, and early childhood, when the organism is highly sensitive to metabolic and nutritional perturbations. Maternal lifestyle and nutritional status are among the most influential determinants of fetal programming, shaping long-term susceptibility to metabolic disorders. Maternal malnutrition (whether undernutrition, overnutrition, or obesity) can induce persistent epigenetic modifications that alter gene expression without changing the DNA sequence. These changes may explain the increased risk of obesity, insulin resistance, and type 2 diabetes observed in offspring exposed to adverse intrauterine environments, such as maternal obesity or GDM. Once established early in life, these epigenetic alterations are relatively stable and may exert lifelong effects on phenotype and disease risk. The main mechanisms implicated include DNA methylation, mediated by DNA methyltransferases (DNMTs), which typically repress gene transcription when occurring in promoter regions; histone modifications (e.g., acetylation, methylation, phosphorylation) that influence chromatin structure and the accessibility of transcriptional machinery; and regulation by microRNAs (miRNAs), small non-coding RNAs that post-transcriptionally regulate gene expression. Together, these mechanisms form the molecular basis of fetal programming, through which maternal nutritional imbalance can imprint lasting effects on gene regulation, metabolism, and disease vulnerability in later life [157,158,159].

In 2018, Hjort. et al. conducted a large study on the offspring DNA methylation of mothers with GDM. The researchers examined the relationship between DNA methylation profiles of 93 children aged between 9 and 16 years and their exposure to GDM during pregnancy [160]. Venous blood analysis showed that 76 CpG sites were differently methylated compared to control subjects (95 offspring of mothers with non-complicated pregnancies). After adjustment for maternal pre-pregnancy BMI, 13 of these sites were identified as closely related to GDM, of which 9 were previously related to metabolic and cardiovascular diseases such as type II diabetes (T2D), obesity, diabetic nephropathy or coronary heart disease; moreover, 20 CpG sites were associated with pre-pregnancy maternal obesity independently of GDM [160].

Hyperglycemia in pregnancy, even when not associated with maternal obesity, seems to be one of the most significant factors in the determination of fetal programming. Most human studies that evaluated the epigenetic mechanisms involved in utero exposure to GDM have identified several differentially methylated genes, including gene loci related to leptin (LEP), adiponectin (ADIPOQ) and the SLC2A1 and SCL2A3 genes, which code respectively for glucose membrane transporters GLUT1 and GLUT3. Furthermore, DNA methylation levels of the key gene PPARGC1α, involved in glucose and fat metabolism that codes for the protein PGC1α, were increased [161,162].

Franzago et al. (2024) investigated the influence of maternal metabolic status on DNA methylation of two key genes involved in energy and lipid metabolism, MC4R and LPL, in a cohort of 101 mother–child pairs [163]. DNA methylation was analyzed in maternal blood, placental tissue, and buccal swabs from offspring born to women with or without obesity and GDM. The majority of participants (≈80%) were diagnosed with GDM, and nearly half were overweight or obese prior to pregnancy [163].

Given the critical role of the melanocortin-4 receptor (MC4R) in regulating appetite and energy balance, and of lipoprotein lipase (LPL) in placental lipid transport, the study examined whether adverse intrauterine environments could epigenetically alter these loci. Results showed that gestational age at delivery was slightly lower among GDM and obese women, MC4R promoter methylation was reduced in newborns of GDM mothers, while placental LPL methylation was increased on the fetal side of obese placentas. Moreover, methylation levels of both genes were negatively correlated with birth weight, suggesting that epigenetic modulation of these pathways may influence fetal growth and metabolic programming [163].

Overall, the findings indicate that maternal obesity and hyperglycemia during pregnancy can induce specific DNA methylation changes in MC4R and LPL, potentially shaping offspring susceptibility to metabolic disturbances later in life [163,164].

Further evidence of the epigenetic influence of maternal metabolic status on offspring was provided by Hjort et al. (2024), who investigated differentially methylated regions (DMRs) in blood from children exposed to GDM compared with controls [165]. The study, which included over 1200 children aged 9–16 years from the Danish National Birth Cohort, identified a significant DMR encompassing the non-coding RNA nc886 (VTRNA2-1) locus—a region known to undergo maternal imprinting in approximately 75% of offspring and considered a metastable epiallele sensitive to periconceptional environmental conditions [165].

Follow-up analyses in an independent cohort of adult offspring (the Copenhagen Offspring Cohort) revealed a conserved bimodal methylation pattern of nc886 across blood, skeletal muscle, and subcutaneous adipose tissue, consistent with its epigenetic metastability. Adults and children exposed to GDM displayed hypomethylation of the nc886 DMR compared with controls, and methylation levels were inversely correlated with nc886 RNA expression, particularly in adipose tissue. Increased nc886 expression in adipose tissue was associated with higher maternal pre-pregnancy BMI, as well as elevated fasting insulin and C-peptide and lower HDL cholesterol in the offspring, suggesting links with adverse cardiometabolic phenotypes.

Collectively, these findings indicate that maternal obesity and GDM may induce long-lasting epigenetic changes at the nc886 locus, potentially altering gene regulation across multiple tissues and contributing to the intergenerational transmission of metabolic risk [165].

**Figure 3:**
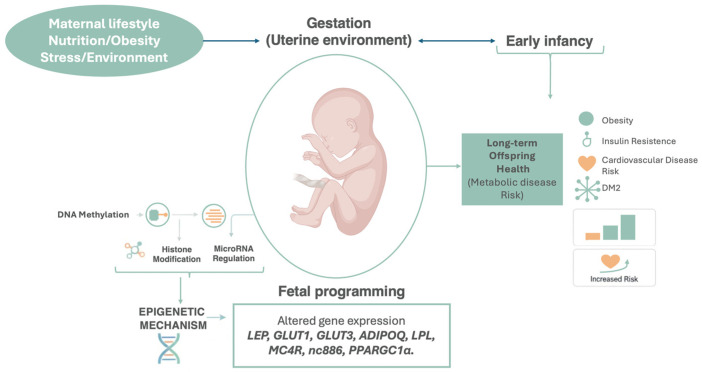
Epigenetic mechanisms and transgenerational transmission of metabolic risk. Maternal lifestyle factors influence the intrauterine environment, inducing epigenetic modifications (DNA methylation, histone modification, and microRNA regulation) that alter metabolic gene expression and program long-term offspring health, increasing the risk of obesity, insulin resistance, type 2 diabetes, and cardiovascular disease. Original figure created by the authors using Microsoft PowerPoint Office 365.

An innovative contribution to the understanding of the epigenetic impact of GDM on offspring was provided by Kanney et al., who explored the relationship between DNA methylation patterns and epigenetic age acceleration in newborns. The study involved 137 mother–child dyads, and biological age was estimated using the Horvath and Hannum epigenetic clocks, which calculate DNA methylation age from 353 to 71 CpG sites, respectively. The authors observed that children exposed to GDM in utero exhibited a stronger correlation between chronological and epigenetic age compared with controls, indicating accelerated biological aging in this group. Furthermore, the analysis of metabolic biomarkers revealed a significant association between fasting insulin levels and epigenetic age acceleration, suggesting a link between intrauterine exposure to GDM and early metabolic alterations. However, this association was no longer significant after adjusting for offspring BMI, indicating that body weight mediates the relationship between GDM exposure and epigenetic aging [166]. These findings reinforce the concept that maternal metabolic conditions may leave a persistent molecular signature on offspring, influencing both metabolic trajectories and biological aging [167,168]. Future studies are needed to determine whether such epigenetic alterations are reversible through early lifestyle or nutritional interventions (Figure 3).

## 7. Malnutrition During Pregnancy, a Transgenerational Problem: Transgenerational Inheritance and the Paternal Role

One of the most emblematic examples of how maternal malnutrition can exert long-lasting and even transgenerational effects is the Dutch Hunger Winter. This famine not only provided the first evidence linking maternal nutrition to offspring health but also revealed that the metabolic consequences of prenatal undernutrition could extend to the second generation. Studies on the grandchildren of women exposed to famine in utero showed that grandsons of women malnourished during early pregnancy tended to have higher birth weights, while exposure in late pregnancy did not affect birth weight [169,170,171,172].

These findings suggest that epigenetic modifications induced by early intrauterine nutrient deprivation can affect the germline (since oocytes develop during fetal life) thereby transmitting altered metabolic regulation across generations. Such changes may also modify placental nutrient transport, promote compensatory nutrient delivery and predispose offspring to metabolic disorders later in life [18,173,174].

The transgenerational effects of maternal overnutrition have similarly been confirmed in experimental models. Masuyama et al. demonstrated in mice that exposure to a high-fat diet (HFD) during pregnancy induces epigenetic alterations in genes encoding the adipokines leptin and adiponectin, key regulators of energy balance and insulin sensitivity. In this multigenerational design, F0 females were fed either an HFD or a control diet before and during gestation; F1 offspring were exposed to the same or opposite diets, and the F2 generation, which was never directly exposed to HFD, was evaluated for metabolic outcomes. Despite the absence of direct dietary exposure, F2 offspring from HFD lineages exhibited increased body weight, adiposity, blood pressure, insulin resistance, and dyslipidemia, with a clear trend in metabolic impairment following the cumulative exposure of previous generations (A > B > C > D) [175]. Molecular analyses confirmed histone modifications in the promoter regions of leptin and adiponectin, including reduced H3K9 acetylation and increased H3K9 demethylation and H4K20 monomethylation, consistent with altered gene transcription and circulating adipokine levels [176].

Together, these findings provide compelling evidence that both maternal undernutrition and overnutrition can induce heritable epigenetic modifications affecting energy metabolism, thereby perpetuating metabolic dysfunction across generations. Such mechanisms underscore the transgenerational nature of nutritional programming and highlight the critical role of maternal metabolic health in shaping disease risk in future generations [169,175].

While much focus has been placed on maternal influences, emerging evidence indicates that paternal factors also contribute to fetal programming through epigenetic modifications in sperm [73,177]. The first indication came from Ng et al., who showed that male mice fed an HFD developed obesity, insulin resistance, and hepatic steatosis, and when mated with control-fed females, their female offspring exhibited impaired glucose tolerance and a reduction in pancreatic β-cell mass, despite normal body weight. This suggested a paternal transmission of metabolic risk independent of maternal environment [178].

Further studies have explored the molecular basis of paternal inheritance, focusing on genes involved in appetite regulation and energy balance, such as POMC, a key component of the hypothalamic melanocortin pathway. In a recent mouse model, Haberman et al. showed that males fed an HFD exhibited differential methylation at 24 CpG sites in the POMC promoter region of sperm DNA, with methylation levels correlating positively with body weight [179]. Offspring of these males displayed altered POMC mRNA expression and higher leptin levels, even when fed a control diet (HF–CH), indicating that the paternal HFD exposure induced stable epigenetic modifications transmitted through sperm. Importantly, methylation changes in the POMC promoter persisted in the sperm of F1 males, confirming a transgenerational inheritance of paternal epigenetic marks [179].

Together, these findings demonstrate that both maternal and paternal nutritional environments before and during conception can profoundly influence offspring metabolic programming through heritable epigenetic mechanisms. The concept of transgenerational inheritance thus expands the traditional view of fetal programming, emphasizing that parental health and diet—not only during pregnancy but also prior to conception—play a crucial role in shaping disease susceptibility across generations [73,169,175,177,178,179].

## 8. Impacts of Lifestyle Interventions

Given the strong evidence linking maternal obesity and dysregulated energy balance during pregnancy to adverse metabolic outcomes in offspring, several studies have investigated whether lifestyle and dietary interventions during gestation could mitigate these effects. However, the results remain largely inconclusive [165]. In a randomized controlled study conducted in Denmark, Tanvig et al. examined the impact of a combined diet and physical activity intervention in obese pregnant women (BMI 30–45 kg/m^2^) on offspring metabolic outcomes up to 2.5–3 years of age. The cohort included 150 mother–child pairs from the Lifestyle in Pregnancy (LiP) study and 97 external controls. Despite improved maternal lifestyle during gestation (LiPi group), no significant benefits were observed in the offspring, with only a modest increase in abdominal circumference compared to controls. Other metabolic markers, including BMI, fasting glucose, insulin, and lipid profile, were not affected [180]. Similarly, Mogensen et al. conducted a randomized controlled trial on 208 obese women (pre-pregnancy BMI 28–45 kg/m^2^), comparing two dietary patterns aimed at controlling gestational weight gain: a high-protein, low-glycemic index (HPLGI) diet versus a moderate-protein, moderate-glycemic index (MPMGI) diet. Offspring were followed up to five years of age, with assessments at birth, 6 months, 18 months, 3 years, and 5 years. No consistent differences emerged between groups in anthropometric or metabolic parameters; however, minor transient alterations were noted—higher glucose and lower insulin levels in cord blood at birth, slightly higher adiposity at 18 months, and a trend toward a more atherogenic lipid profile (reduced HDL, elevated triglycerides, total cholesterol, and LDL) in the HPLGI group at later follow-ups [181].

Overall, the lack of long-term improvement in offspring metabolic outcomes suggests that interventions limited to pregnancy may be insufficient to counteract the epigenetic and metabolic consequences of maternal obesity. Instead, both studies underscore the critical importance of preconception health optimization, emphasizing that maternal BMI before pregnancy is a stronger determinant of offspring metabolic risk than gestational lifestyle changes alone [180,181].

Other recent clinical evidence (2023–2025) has extended the range of lifestyle interventions studied in pregnancy. For example, individualized exercise guidance reduced macrosomia and large-for-gestational-age births, mediated by gestational weight gain, but did not report long-term offspring metabolic outcomes [182].

The FitMum trial and pedometer-based activity monitoring demonstrate that enhanced physical activity can favorably influence GWG and some perinatal outcomes, though effects on maternal metabolic health and child outcomes remain minimal or uncertain [183].

In the DALI lifestyle intervention, combined diet and activity counseling resulted in attenuated fetal growth trajectories in male offspring, highlighting potential sex-specific responses to interventions [184].

Interestingly, maternal adherence to a Mediterranean diet during pregnancy has been associated with a lower risk of overweight/obesity at 24 months in offspring, suggesting that dietary quality may exert early programming effects [185].

Nevertheless, larger pooled analyses reinforce that antenatal interventions alone show limited durability in improving offspring cardiometabolic risk, underscoring the importance of optimizing health before conception [186].

## 9. Conclusions

Parental nutrition, encompassing both maternal and paternal contributions, profoundly influences offspring health through metabolic and epigenetic pathways. Within the DOHaD framework, the intrauterine environment represents a critical window during which nutritional signals direct fetal growth, cellular metabolism, and long-term gene regulation. Maternal overnutrition, obesity, and GDM activate anabolic and inflammatory pathways, leading to excessive nutrient transfer and predisposing offspring to obesity and insulin resistance. In contrast, undernutrition and micronutrient deficiencies disrupt organ development and trigger adaptive metabolic responses that may become maladaptive postnatally. Maternal obesity impacts offspring health both in the short and long term, altering newborn body composition and fat distribution, thereby increasing risks for cardiovascular disease, cancer, and mortality in adulthood. Epigenetic modifications—including DNA methylation, histone remodeling, and microRNA dysregulation—underpin these effects and may be transmitted across generations. Nutritional imbalances in both parents can induce heritable changes in gene expression governing energy balance and metabolism, perpetuating disease susceptibility.

**Figure 4:**
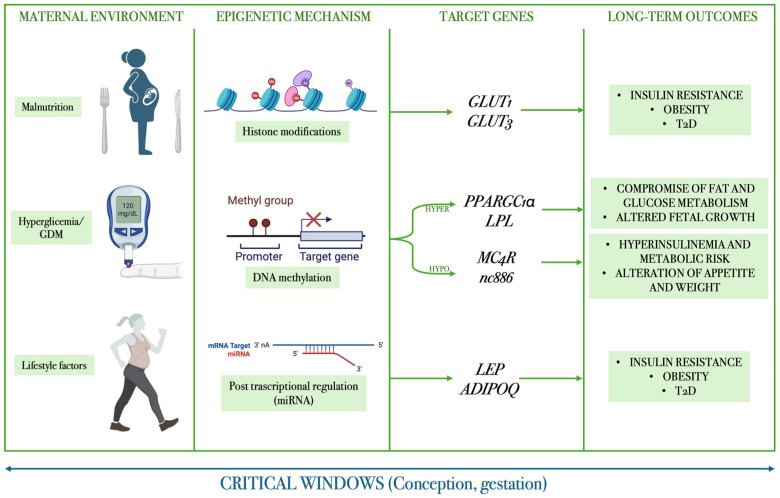
Diagram depicting how maternal lifestyle factors influence the intrauterine environment, inducing epigenetic modifications (DNA methylation, histone modification, and microRNA regulation). Specific epigenetic marks regulate metabolic gene expression and influence long-term outcomes and transgenerational transmission of metabolic risk, increasing the risk of obesity, insulin resistance, type 2 diabetes, and cardiovascular disease. Original figure created by the authors using Microsoft PowerPoint Office 365.

Importantly, the preconception period emerges as a fundamental window of intervention that has been historically underappreciated. Optimizing nutrition in both prospective mothers and fathers before conception can establish favorable epigenetic landscapes, normalize gamete quality, and prevent the transmission of metabolically compromised phenotypes. Paternal nutrition, in particular, warrants greater clinical and research attention, as emerging evidence demonstrates that paternal obesity, micronutrient deficiencies, and metabolic dysfunction can independently alter sperm epigenome and offspring metabolic health, extending beyond the traditional maternal-centric paradigm (Figure 4).

Future research should prioritize several key directions: (I) Characterizing paternal nutritional contributions through longitudinal studies examining how paternal diet quality, obesity, and micronutrient status influence sperm epigenetics and offspring outcomes across multiple generations. (II) Defining critical preconception nutritional windows for both parents, identifying optimal duration and composition of dietary interventions to maximize beneficial epigenetic programming. (III) Developing evidence-based preconception nutritional guidelines that integrate both macronutrient balance and micronutrient optimization for couples planning pregnancy. (IV) Investigating epigenetic reversibility and intervention strategies to determine whether and how nutritional or pharmacological approaches can restore healthy epigenetic marks in already compromised lineages.

Thus, nutrition serves not only as an energy source but as a biological signal programming health trajectories throughout life. Ensuring optimal maternal and paternal nutrition in the preconception period and during pregnancy represents a vital intergenerational preventive strategy to reduce the global burden of metabolic and chronic diseases.

## Data Availability

No new data were created or analyzed in this study.

## Abbreviations

The following abbreviations are used in this manuscript:

A2BAR	A2B Adenosine Receptor
ACTH	Adrenocorticotropic Hormone
ADIPOQ	Adiponectin gene
ARH	Arcuate nucleus of the hypothalamus
BDI	Beck Depression Inventory
BIA	Bioelectrical Impedance Analysis
BMI	Body Mass Index
CHD	Coronary Heart Disease
CpG	Cytosine–Phosphate–Guanine site
CRH	Corticotropin-Releasing Hormone
CVD	Cardiovascular Disease
DNMT	DNA Methyltransferase
DHEAS	Dehydroepiandrosterone Sulfate
DMR	Differentially Methylated Region
F0/F1/F2	Generazioni in modelli animali
FT3	Free Triiodothyronine
FT4	Free Thyroxine
GH	Growth Hormone
GLP-1	Glucagon-Like Peptide-1
GLUT1	Glucose Transporter 1
GLUT3	Glucose Transporter 3
HAIR	Hyperinsulinemia Androgenism Insulin Resistance
HbA1c	Hemoglobin A1c
HDRS	Hamilton Depression Rating Scale
HFD	High-Fat Diet
HOMA-IR	Homeostatic Model Assessment of Insulin Resistance
HPLGI	High-Protein Low-Glycemic Index diet
Hs-CRP	High-sensitivity C-Reactive Protein
HPA axis	Hypothalamic–Pituitary–Adrenal axis
IC M	Insufficienza Cardiaca Metabolica
IFG	Impaired Fasting Glucose
IGF	Insulin-like Growth Factor signaling
IGF-1	Insulin-like Growth Factor 1
IGT	Impaired Glucose Tolerance
IL-6	Interleukin-6
ISI	Insulin Sensitivity Index
IVGTT	Intravenous Glucose Tolerance Test
LPL	Lipoprotein Lipase
MACE	Major Adverse Cardiovascular Events
MI	Myocardial Infarction
miRNA	microRNA
MPMGI	Moderate-Protein Moderate-Glycemic Index diet
mTOR	Mammalian Target of Rapamycin
NAFLD	Non-Alcoholic Fatty Liver Disease
nc886 (VTRNA2-1)	Non-coding RNA locus/metastable epiallele
OCs	Oral Contraceptives
OGTT	Oral Glucose Tolerance Test
PCS	Pain Catastrophizing Scale
PCEs	Pooled Cohort Equations
PGC1α/PPARGC1α	Peroxisome proliferator-activated receptor gamma coactivator 1-alpha
POMC	Proopiomelanocortin
QCA	Quantitative Coronary Angiography
RMN	Risonanza Magnetica Nucleare
SHBG	Sex Hormone-Binding Globulin
SLC2A1	Gene codificante GLUT1
SLC2A3	Gene codificante GLUT3
SNS	Sympathetic Nervous System
SSRI	Selective Serotonin Reuptake Inhibitors
STAI	State-Trait Anxiety Inventory
STZ	Streptozotocin
T2D	Type 2 Diabetes
TNF-α	Tumor Necrosis Factor alpha
TSH	Thyroid-Stimulating Hormone
VAT	Visceral Adipose Tissue
WAT	White Adipose Tissue
WHR	Waist-to-Hip Ratio
